# *Xenopus* Pkdcc1 and Pkdcc2 Are Two New Tyrosine Kinases Involved in the Regulation of JNK Dependent Wnt/PCP Signaling Pathway

**DOI:** 10.1371/journal.pone.0135504

**Published:** 2015-08-13

**Authors:** Marta Vitorino, Ana Cristina Silva, José Manuel Inácio, José Silva Ramalho, Michal Gur, Abraham Fainsod, Herbert Steinbeisser, José António Belo

**Affiliations:** 1 Regenerative Medicine Program, Departamento de Ciências Biomédicas e Medicina, Universidade do Algarve, Faro, Portugal; 2 Center for Biomedical Research (CBMR), Universidade do Algarve, Campus de Gambelas, Faro, Portugal; 3 Department of Developmental Biology and Cancer Research, Institute for Medical Research Israel-Canada, Faculty of Medicine, Hebrew University, P.O. Box 12272, Jerusalem, 91120, Israel; 4 Institute of Human Genetics, University of Heidelberg, Heidelberg, Germany; 5 CEDOC, NOVA Medical School/Faculdade de Ciências Médicas, Universidade Nova de Lisboa, Lisboa, Portugal; CHU Sainte Justine and University of Montreal, CANADA

## Abstract

Protein Kinase Domain Containing, Cytoplasmic (PKDCC) is a protein kinase which has been implicated in longitudinal bone growth through regulation of chondrocytes formation. Nevertheless, the mechanism by which this occurs remains unknown. Here, we identified two new members of the PKDCC family, Pkdcc1 and Pkdcc2 from *Xenopus laevis*. Interestingly, our knockdown experiments revealed that these two proteins are both involved on blastopore and neural tube closure during gastrula and neurula stages, respectively. In vertebrates, tissue polarity and cell movement observed during gastrulation and neural tube closure are controlled by Wnt/Planar Cell Polarity (PCP) molecular pathway. Our results showed that Pkdcc1 and Pkdcc2 promote the recruitment of Dvl to the plasma membrane. But surprisingly, they revealed different roles in the induction of a luciferase reporter under the control of *Atf2* promoter. While Pkdcc1 induces *Atf2* expression, Pkdcc2 does not, and furthermore inhibits its normal induction by Wnt11 and Wnt5a. Altogether our data show, for the first time, that members of the PKDCC family are involved in the regulation of JNK dependent Wnt/PCP signaling pathway.

## Introduction

PKDCC (Protein Kinase Domain Containing, Cytoplasmic) is a protein from a novel family of Serine/Tyrosine/threonine kinase catalytic domain proteins, which localizes in the Golgi complex and whose function was recently proposed in the mouse [1–3]. During embryonic development of mice, PKDCC (also referred as VLK and ADTK) was described to be involved in protein export from the Golgi, and to be essential for stromal function of mesenchymal cells [3]. In mouse embryos, the absence of *Pkdcc* leads to the development of small animals, with cranial abnormalities, deficient long bone elongation due to a delay in flat proliferative chondrocyte formation, sternal dysgraphia, shortened intestine, cleft palate and lung hypoplasia. In addition, the newborn knockout mice die a few hours after birth due to abnormal respiration [1–3].

Despite these phenotypes described on the knockout of *Pkdcc* in mouse embryos [1–3], the mechanism by which this gene function is completely unknown. Probst *et al* proposed a genetic interaction between *Pkdcc* and *Gli3* during mouse development. The authors suggest that both *Pkdcc* and *Gli3* cooperate on the regulation of long bone formation by modulating the temporal kinetics of columnar and hypertrophic chondrocyte domains establishment [4]. Nevertheless, they presented an alternative model where *Pkdcc* could also modulate Wnt signaling, since inactivation of *Wnt5a* also alters the transition between proliferating to hypertrophic chondrocytes [4]. They suggest that, since Pkdcc regulates protein export from Golgi [3], its inactivation may directly interfere with either the secretion of the relevant signals or cell-surface localization of receptors [4].

Cell movements are essential for the correct shape of body axis and organ formation during embryo development. These morphogenetic cell movements are not stochastic, they undergo extensive control by distinct signal transduction pathways. One of this pathways is Wnt/Planar Cell Polarity (PCP) signaling pathway that, for example, in polarised tissue, coordinate the morphogenetic processes of the cells in the epithelial sheets plane. [5]. A set of core proteins was identified to be involved in PCP pathway, in both vertebrates and invertebrates. In vertebrates this group include the transmembrane receptor Fizzled (Fz), the cytoplasmic molecules Dishevelled (Dvl), Diego (Dgo) and Prickle (Pk), the transmembranar protein VanGogh/Strabismus (Vang/Stbm) and the cadherin-like protein Flamingo/Celsr1 (Fmg/Clsr1). These core PCP components were identified as genes whose inactivation leads to cell polarity mis-alignment [6–8]. The PCP is involved in the coordination of cells within a tissue sheet, either by direct cell-cell interaction [7, 8] or under the influence of a diffusible ligand-based signalling system [9]. This occurs because these proteins localize in different regions inside the cell: Fz, Dvl and Dgo are localized in the proximal region, Vangl2 and Pk in distal region and Clsr1 localize in both distal and proximal regions, which is essential for the proper establishment of polarization [5, 10, 11].

It has been shown that alterations in the different PCP pathway proteins lead to various diseases, including developmental ones like neural tube defects [12]. For example, mice, frog and zebrafish embryos with defective Vangl2 display neural tube defects [13–15]. Dvl2 knockout mice also displayed thoracic spina bifida [16], and several point mutation were identified in genes of PCP pathway in humans that display a sort of neural tube defects [17, 18].

Here, we described the role of two novel kinase proteins from PKDCC family, Pkdcc1 and Pkdcc2, during *Xenopus laevis* embryonic development. We describe the expression pattern of both genes, and we show that the absence of both proteins during early development induces a delay in blastopore and neural tube closure. We present the first evidences that some PKDCC family members are involved in the regulation of PCP signaling pathway.

## Materials and Methods

### Ethics statement

The studies involving animal experiments are in accordance to the ethical issues for clinical research and EU guidelines for animal research. All animal work performed in this study was conducted in compliance with the Portuguese law and approved by the Consultive Commission of the Veterinary Agency from Portuguese Ministry of Agriculture (Directive 2010/63/EU of the European Parliament), the Agency responsible for issuing approval for experimental animal studies, following the EU guidelines for animal research and welfare.

### *X*. *laevis* embryo manipulations

*X*. *laevis* eggs were obtained from females and manipulated as previously described [19] and staged according to Nieuwkoop and Faber [20].

### Cloning of Xenopus pkdcc1 and pkdcc2

*X*. *laevis pkdcc1* and *pkdcc2* were identified by using the translated nucleotide sequence of the mouse *Pkdcc* as queries to perform TBLASTX comparisons against NCBI’s translated nucleotide (nt) and EST databases (dbest). Protein sequence alignments and homology scores were derived from NCBI’s BL2SEQ alignment program. SMART (http://smart.embl-heidelberg.de/) and PHI-BLAST (Pattern Hit Initiated BLAST) bioinformatic tools were used to analyse the domain arquitecture of the proteins. *X*. *laevis* EST containing the full open reading frame of *pkdcc2* (Genbank accession number: BJ630561) was obtained from NIBB (http://Xenopus.nibb.ac.jp/).

No *pkdcc1* full open reading frame EST clone was found in any of the search databases. In addition, no partial coding sequence *pkdcc1* clone was retrievable from the stock centers but was kindly gifted by M. Taira (Genbank accession number: BP673009). To isolate the full length coding sequence, total RNA from *X*. *laevis* gastrula stage embryos [20] was isolated using Trizol reagent (Invitrogen) according to the manufactures protocol. First strand cDNA was synthesized with H-Minus M-MulV reverse transcriptase (Fermentas) using random hexamers as primers. The *pkdcc1* was amplified by PCR using a specific pair of primers (S1 Table) and introduced into pCS2^+^ plasmid.

### Axis perturbation assay

To perform UV and LiCl treatments the embryos were treated as described by Sive *et al* [21]. After the first cleavage occurred, the embryos were transferred to 0.1XMBS-H (1XMBS-H = 88 mM NaCl, 1 mM KCl, 2.4 mM NaHCO_3_, 0.82 mM MgSO_4_, 0.41 mM CaCl_2_, 0.33 mM Ca(NO_3_)_2_, 10 mM HEPES pH 7.4, 10 μg/ml streptomycin sulfate and 10 μg/ml penicillin) agarose coated dishes and allowed to grow at 20°C, at the same time as untreated embryos. At stage 10.5, the embryos were fixed in MEMFA (0.1 mM MOPS pH7.4, 2 mM EGTA, 1 mM MgSO_4_, 3.7% formaldehyde) and stored.

### mRNA synthesis and microinjection

Capped sense mRNAs were synthesized using the Ambion mMessage mMachine kit (*Applied Biosystems*). *In vitro* fertilization and microinjection of *X*. *laevis* embryos were performed as previously described [22].

### Whole mount *in situ* hybridization and histology

Single and double whole mount *in situ* hybridization and anti-sense probe preparation was carried out as previously described [23, 24]. To generate the digoxigenin labelled *Xbra*, *pkdcc1* and *pkdcc2* antisense RNA probes, plasmids containing *Xbra*, *pkdcc1* and *pkdcc2* fragments were linearized using *EcoRV*, *SalI* and *EcoRI* restriction enzymes and transcribed using T7, T3 and T7 RNA polymerases, respectively. To generate the fluorescein labelled *otx2* and *cardiac troponin* antisense RNA probes, plasmids containing *otx2* and *cardiac troponin* fragments were linearized using *EcoRI* and *NotI* restriction enzymes and transcribed using T3 and T7 RNA polymerases, respectively. After *in situ* hybridization, stained embryos were bleached by illumination in 1% H_2_O_2_, 4% formamide and 0.5X SSC pH7.0. Embryos were photographed under bright light using a MicroPublisher 5.0 RTV camera coupled with a Leica MZ16FA stereoscope.

### Plasmid constructs and morpholino Oligonucleotide

The *X*. *laevis pkdcc1* and *pkdcc2* morpholino oligonucleotides (pkdcc1Mo and pkdcc2Mo, respectively) were synthesized and obtained from Gene Tools LLC. pkdcc1Mo was designed to complement region between AUG and +25 downstream of the AUG (5’-CGCACAGGCTAATGGTGTTCTTCAT-3’), whereas pkdcc2Mo was designed to complement region between base -1 upstream of the AUG and base +24 downstream of the AUG (5’-CACTGCGATCTTCCTGCGTCTCATG-3’). The standard control morpholino oligonucleotide was the following (5’-CCTCTTACCTCAGTTACAATTTATA-3’)

To test the localization of Pkdcc1 and Pkdcc2 proteins, two C-terminal tagged constructs were generated. The Pkdcc1-HA that contains the entire *X*. *laevis pkdcc1* CDS fused with an HA tag. This construct was generated by digestion and sub-cloning of *pkdcc1* fragment on pBSII(SK). To mutate the stop codon, a pair of oligonucleotides (Pkdcc1HA; S1 Table) were annealed in annealing buffer (100mM potassium acetate, 30mM HEPES-KOH pH 7.4, 2mM Mg-acetate), 4 min at 95°C, followed by 10 min at 70°C and slowly cooled down to 4°C. The annealed oligonucleotides were subcloned in the anterior plasmid resulting in pBSII(SK).Pkdcc1. This plasmid was digested and cloned into pCS2^+^.3HA.

The Pkdcc2-myc that contain *X*. *laevis pkdcc2* complete CDS plus the 1bp upstream the ATG fused with a myc tag. This construct was generated by PCR amplification of pBSII(SK).Pkdcc1 to mutate the stop codon. For that, we used a pair of primers described in Pkdcc2myc; S1 Table. The PCR product was cloned into PGEM-T easy (Promega) and then subcloned into pCS2^+^.6xmyc.

In order to generate pENTR-GFP-Rab8a, a mammalian expression Gateway (Invitrogen) vector was used. pENTR-GFPC2 was generated based on pENTR-V5 [25], by swapping part of the CMV promoter, V5 tag and the polylinker with the equivalent sequences containing GFP tag sequence from pEGFPC2 (Clontech), using NdeI/BamHI restriction sites. Rab8a murine coding sequence and part of 3’ UTR were produced by RT-PCR amplification (forward primer- 5’–AGTGAATTCATGGCGAAGACGTACGATTATCTGTTC -3’; and reverse primer- 5’–catgtcgacaacagcaaaattctaactctctccatc– 3’) using total RNA isolated from at-T20 cell line as a template, digested with EcoRI/SalI and cloned into pENTR-GFPC2 with the same restriction enzymes.

### Animal caps elongation assay

The embryos were injected at 2 to 4 cell stage in the animal pole. The animal caps were extracted at stage 8 and cultured in 1x Steinberg solution with or without 10ng/mL of activin. The explants were grown until sibling embryos raised stage 15.

### Dishevelled localization

To localize Dishevelled protein inside *X*. *laevis* animal cap cells it was used RNA synthesized from a plasmid containing the *dvl* coding sequence fused with a GFP reporter, *dvl*.*GFP* or from a plasmid containing the DEP domain from *dvl* sequence tagged with a GFP reporter, *DEP*.*dvl* [26]. The animal caps were extracted at stage 10 [20] fixed in MEMFA and observed in a Zeiss LSM710 confocal microscope.

### Luciferase assay

To analyse JNK activity (PCP pathway) in the presence of Pkdcc1 and Pkdcc2, HEK293T cells were transfected with 100 ng *Atf2* luciferase reporter and 10 ng of *β-galactosidase* (*β-gal*) in combination with 500 ng of *wnt11*, 500 ng of *wnt5a*, 1 μg of *pkdcc1* or 1 μg of *pkdcc2* DNA plasmids. The cells were allowed to grow for 48 h. Cell lysates were prepared and luciferase activity was measured using luciferin (Biosynth). β-galactosidade activity was measured using ONPG reagent and used for standardization.

To analyse JNK activity in vivo, *X*.*laevis* embryos were injected at 2 to 4 cell stage with 500 pg of wnt11, 500 pg of wnt5a, 1 ng pkdcc1 or 1 ng of pkdcc2 mRNAs. The embryos were grown until stage 11. Embryo lysates were prepared and luciferase activity was measured using luciferin β-galactosidade activity was measured using ONPG reagent and used for standardization.

### Quantitative PCR

Total RNA from pools of 10 animal caps at stage 11 was isolated using Trizol Reagent (Invitrogen), according to the manufacturer’s instructions. RNA quality analysis and qPCR was performed as described in Perestrelo *et al* [27]. In S2 Table contains a brief description of the primers. Uninjected animal caps were used as negative control and wild type embryos as positive control. Gene expression was normalized to *gapdh* and *odc* expression. All samples and controls were run in triplicate and in three independent biological replicates. Data were presented as the mean and standard error of the mean (SEM).

For elongation assay, total RNA of animal caps were extracted using PerfectPure RNA Tissue Kit with DNase (5 Prime), according to the manufacturer’s instructions. RNA quality analysis and qPCR was performed as described in Perestrelo *et al* [27]. cDNA was synthetized with iScript cDNA Synthesis Kit (Bio-Rad) and qPCR performed in CFX384 Real Time SYStem (Bio-Rad) with Light Cycler 480 SYBR Green I Master (Roche). Gene expression was normalized to *gapdh* expression. All samples and controls were run in triplicate. Data were presented as the mean and standard error of the mean (SEM). In S2 Table contains the primer sequences.

For *pkdcc1* and *pkdcc2* temporal expression, pools of 5 embryos at different developmental stages were isolate using PerfectPure RNA Tissue Kit with DNase (5 Prime), according to the manufacturer’s instructions. RNA quality analysis and qPCR was performed as described in Perestrelo *et al* [27]. cDNA was synthetized with iScript cDNA Synthesis Kit (Bio-Rad) and qPCR performed in CFX384 Real Time SYStem (Bio-Rad) with Light Cycler 480 SYBR Green I Master (Roche) with the primers described in S2 Table. Gene expression was normalized to *gapdh* expression. All samples and controls were run in triplicate. Data were presented as the mean and standard error of the mean (SEM).

### Immunofluorescence

Immunofluorescence of HEK293T cells was performed as previously described [27]. For primary antibodies were used rabbit anti-HA (H6908, Sigma) and mouse anti-myc (9E10, Santa Cruz) and as secondary antibodies were used 594 donkey anti-mouse (Jackson ImmunoReserch) and 594 goat anti-rabbit (Invitrogen). Images were acquired in a Zeiss LSM710 confocal microscope.

## Results

### Expression pattern of *pkdcc1* and *pkdcc2* during early embryogenesis

**Fig 1:**
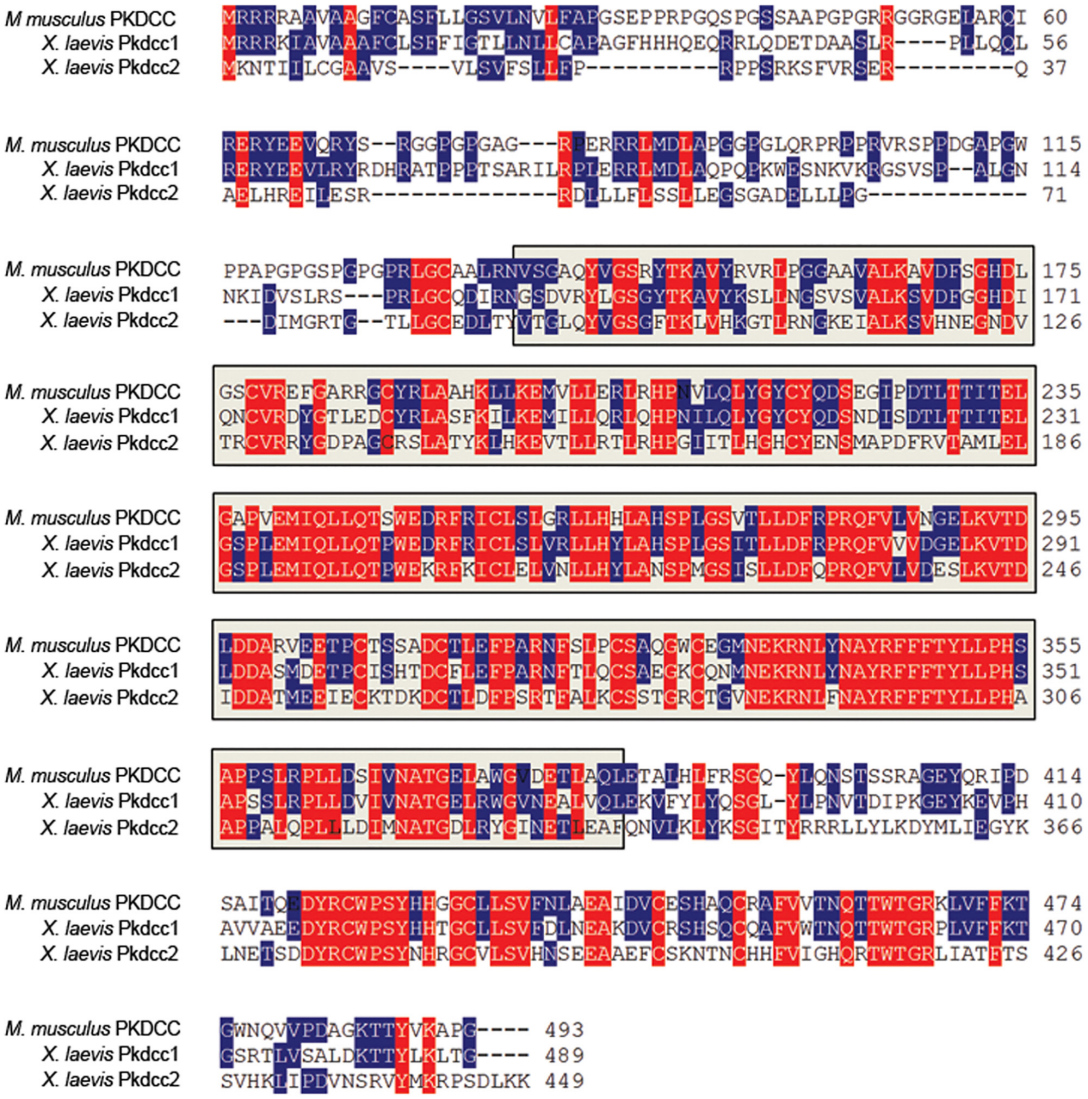
PKDCC protein family encode for a Serine/threonine/tyrosine protein kinase catalytic domain. Comparison of the predicted amino acid sequence of mouse (*M*. *musculus*) PKDCC with its *X*. *laevis* orthologs, Pkdcc1 and Pkdcc2. *pkdcc1* encodes a 449 a.a. protein with a predicted molecular mass of 51.0 kDa and *pkdcc2* encodes for a protein with 489 a.a. and 55.9 kDa of predicted molecular mass. Bioinformatic analysis showed that both proteins contain the Serine/Threonine/Tyrosine protein kinase catalytic domain (between a.a. 89 and 336 for Pkdcc1 and between a.a 134 and 381 for Pkdcc2; Grey box, STYKc domain). Identical amino acids among all are shown in red while identical amino acids in only two sequences are shown in blue. The absence of residues at the corresponding region is indicated by dashes.

We used the sequence of mouse *Pkdcc* (GenBank accession number: NM_134117) to identify two potential *Xenopus laevis* orthologs referred herein as *X*. *laevis pkdcc1* (*protein kinase domain containing*, *cytoplasmic homolog*, *gene 1*; GenBank accession number: NP_001091231) and *X*. *laevis pkdcc2* (*protein kinase domain containing*, *cytoplasmic homolog*, *gene 2*; GenBank accession number: KM 245578) (Fig 1). Bioinformatic analysis (http://smart.heildelberg.de) of Pkdcc1 and Pkdcc2 showed that Pkdcc1 shares 42.0% of identity with its mouse orthologs being the predicted Serine/Threonine/Tyrosine kinase catalytic domain (STYkc), the region of higher identity sharing 57.5% identity. Pkdcc2 has 61.9% of identity with its mouse orthologs while the STYkc of both proteins share 75% of identity. Both *X*. *laevis* proteins share 40.9% identity through the overall protein and 57.8% identity in the region predicted to be protein kinase catalytic domain.

**Fig 2:**
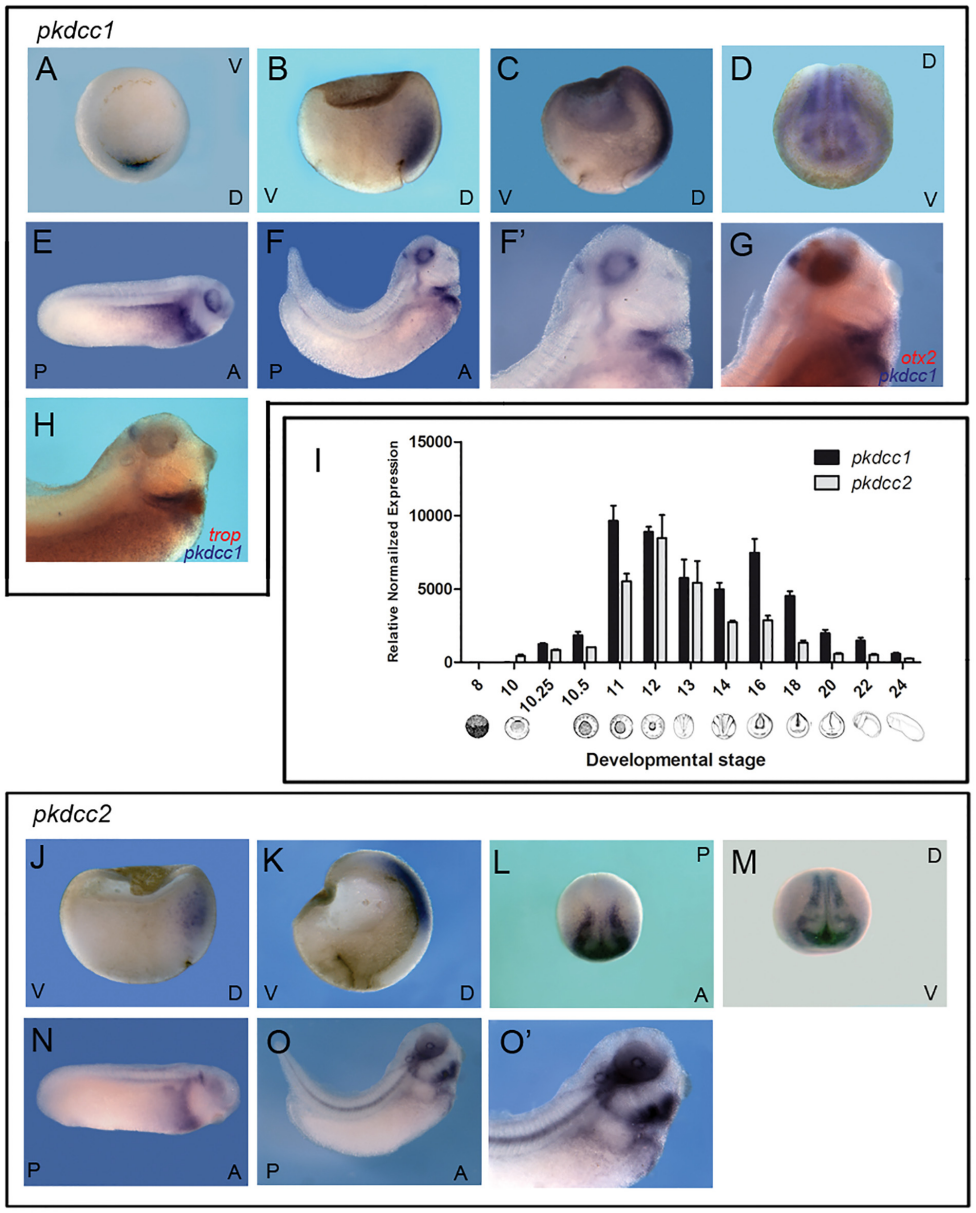
*pkdcc1* and *pkdcc2* expression patterns during *X*. *laevis* embryonic development. (A-H) Expression pattern of *pkdcc1* during early development. (I) Temporal expression pattern of *Xenopus pkdcc1* and *pkdcc2* analysed by quantitative PCR. GAPDH was used as reference gene. (J-O’) Expression pattern of *pkdcc2* during *Xenopus* development. By early gastrula stages, *pkdcc1* transcripts can be detected in the (A) dorsal blastopore lip and (B) ADE. (C) At stage 12, *pkdcc1* is present in the involuting mesoderm. (D) During neurula stages *pkdcc1* is expressed in neural folds and eye fields. (E-F’) *pkdcc1* expression at tailbud stages is restricted to the lateral plate mesoderm, foregut, eye and isthmus. (G) Double whole mount *in situ* hybridization for *pkdcc1* (blue) and *otx2* (red) shows that *pkdcc1* expression in the brain is restricted to the mid-hindbrain boundary. (H) Double whole mount *in situ* hybridization for *pkdcc1* (blue) and *cardiac troponin* (red) showed that *pkdcc1* expression is absent on the heart. (J) Zygotic *pkdcc2* is detectable at early gastrula stages in the anterior dorsal endoderm. (K) At late-gastrula stages *pkdcc2* mRNA is expressed in the involuting mesoderm. (L) During early neurula stages, *pkdcc2* transcripts can be detected in the neural folds (dorsal view, anterior down). (M) At stage 17, *pkdcc2* is co-expressed with *pkdcc1* in the prospective eye field and neural folds. (N-O’) In tailbud stages (27 and 31; lateral view, anterior to the right and dorsal up), *pkdcc2* is expressed in the neural tube roof, otic vesicle, notochord, eye, lateral plate mesoderm and head mesenchyme. D, dorsal; V, ventral; A, anterior; P, posterior.

To analyse the expression pattern of *pkdcc1* and *pkdcc2*, whole-mount *in situ* hybridization and quantitative PCR were performed on *X*. *laevis* embryos at different stages of development. The results showed that *pkdcc1* expression was first detected at early gastrula, stage 10. We observed a first peak at stage 11, a second peak at neurula stage 16 and a decline after that. (Fig 2I). The expression of *pkdcc2* starts at stage 10, in the beginning of gastrula, peaked at stage 12 and started to decrease after that (Fig 2I). At the beginning of gastrulation, both *pkdcc1* and *pkdcc2* are expressed in the anterior dorsal endoderm (ADE; Fig 2B and 2J) being *pkdcc1* also expressed in the dorsal blastopore lip (Fig 2A). As gastrulation proceeds, *pkdcc1* and *pkdcc2* mRNAs are detected not only in the ADE, but also in the involuting dorsal mesoderm, including the prospective prechordal plate (Fig 2C and 2K). During neurula stages, *pkdcc1* is co-expressed with *pkdcc2* in the prospective eye field and in the neural folds (Fig 2D, 2L and 2M). At early tailbud stages (Fig 2E), *pkdcc1* expression can be detected in the lateral plate mesoderm, as well as, in the pronephros, notochord and in the eye. Later, *pkdcc1* expression is restricted to the foregut, notochord and head region (Fig 2F and 2F’). Moreover, a double *in situ* hybridization for *pkdcc1* and *otx2*, (a fore-midbrain marker [28]) showed that *pkdcc1* expression in the brain is posterior to *otx2* expression, confirming *pkdcc1* expression in the isthmus (Fig 2G). Moreover, comparing *pkdcc1* and *cardiac troponin* (cardiac marker [29]) expression patterns during late tailbud stages, we observed that they do not co-localize and thus, *pkdcc1* is not expressed in the heart, but on a close domain, probably the second heart field (Fig 2H). Moreover, the expression of *pkdcc2*, at these tailbud stages, is restricted to the eye, otic vesicle, neural tube roof, notochord, lateral plate mesoderm and head mesenchyme (Fig 2N–2O’).

In summary, even though at later stages *pkdcc1* and *pkdcc2* are expressed in different tissues, they are co-expressed in the ADE, during gastrulation. This data, together with the fact that *Pkdcc* is expressed in the mouse AVE [1–3], the topological equivalent of the frog ADE [23, 30, 31], suggests that this novel gene family has conserved its expression pattern through evolution.

### *pkdcc1* and *pkdcc2* expression is downstream of Wnt canonical signaling

**Fig 3:**
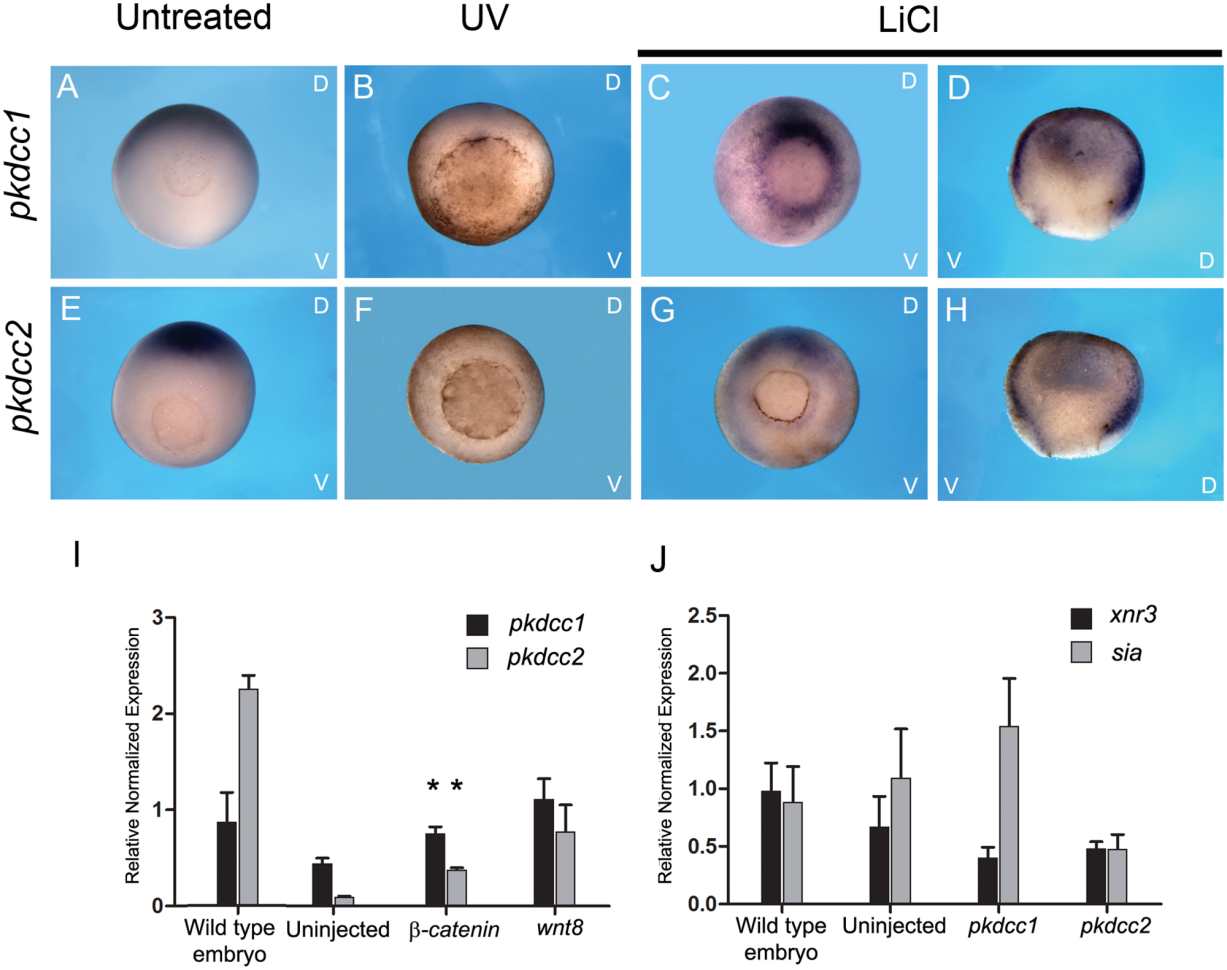
Expression of both *pkdcc1* and *pkdcc2* is induced downstream of Wnt canonical signaling. (A-H) Axis perturbation assay. Whole-mount *in situ* hybridization of gastrula stages untreated embryos (A, E) or treated with UV (B, F) or LiCl (C, D, G, H) and hybridized with (A-D) *pkdcc1* or (E-H) *pkdcc2* probe. Embryos are shown in a vegetal view with the dorsal side at the top and ventral side at the bottom. Hemisections are displayed with the dorsal side to the right. (I) qPCR for *pkdcc1* and *pkdcc2* expression on wild type embryos and uninjected and *β-catenin* or *wnt8* injected animal caps. (J) qPCR analysis of *xnr3* and *siamois* (*sia*) expression on wild type embryos and uninjected and *pkdcc1* or *pkdcc2* injected animal caps. D, dorsal; V, ventral. (p<0.05, in the Student’s *t*-test).

To test if these genes are regulated by signals involved in the specification of dorso-ventral axis, the expression pattern of *pkdcc1* and *pkdcc2* were monitored by whole-mount *in situ* hybridization on UV or LiCl treated embryos and untreated control embryos. The results showed that, in gastrula stage embryos submitted to UV treatment (ventralized embryos), the mRNA levels of *pkdcc1* and *pkdcc2* were greatly reduced (Fig 3, compare A with B, and E with F, respectively). In contrast, in dorsalized embryos obtained by treatment with LiCl, we observe an expansion of *pkdcc1* and *pkdcc2* expression (Fig 3C, 3D, 3G and 3H, respectively). LiCl acts through the inhibition of GSK-3β, allowing the activation of Wnt canonical pathway, required for dorsal axis formation [32]. To further confirm that Wnt canonical induces the expression of both *pkdcc1* and *pkdcc2*, we overexpressed *wnt8* (a morphogen that activates Wnt canonical signaling) and *β-catenin* (a protein that forms a complex with TCF/LEF transcription factors when Wnt canonical is active) in animal caps and the expression of each *pkdcc* gene was analysed by qPCR. The results indicated that both *pkdcc1* and *pkdcc2* are expressed downstream of *β-catenin* and *wnt8*, which are signals involved in the dorsal ventral specification (Fig 3I). In contrast, when both *pkdcc1* and *pkdcc2* were overexpressed in animal caps, the Wnt canonical signaling downstream target genes *Xnr3* and *Sia*, were not upregulated (Fig 3J).

### Pkdcc1 and Pkdcc2 depletion disrupts neural tube closure

To understand the endogenous function of *pkdcc1* and *pkdcc2* during early *X*. *laevis* development, we designed morpholino antisense oligonucleotides (Mo) to knockdown their protein synthesis in the embryo (pkdcc1Mo and pkdcc2MO, respectively) [33, 34].

**Fig 4:**
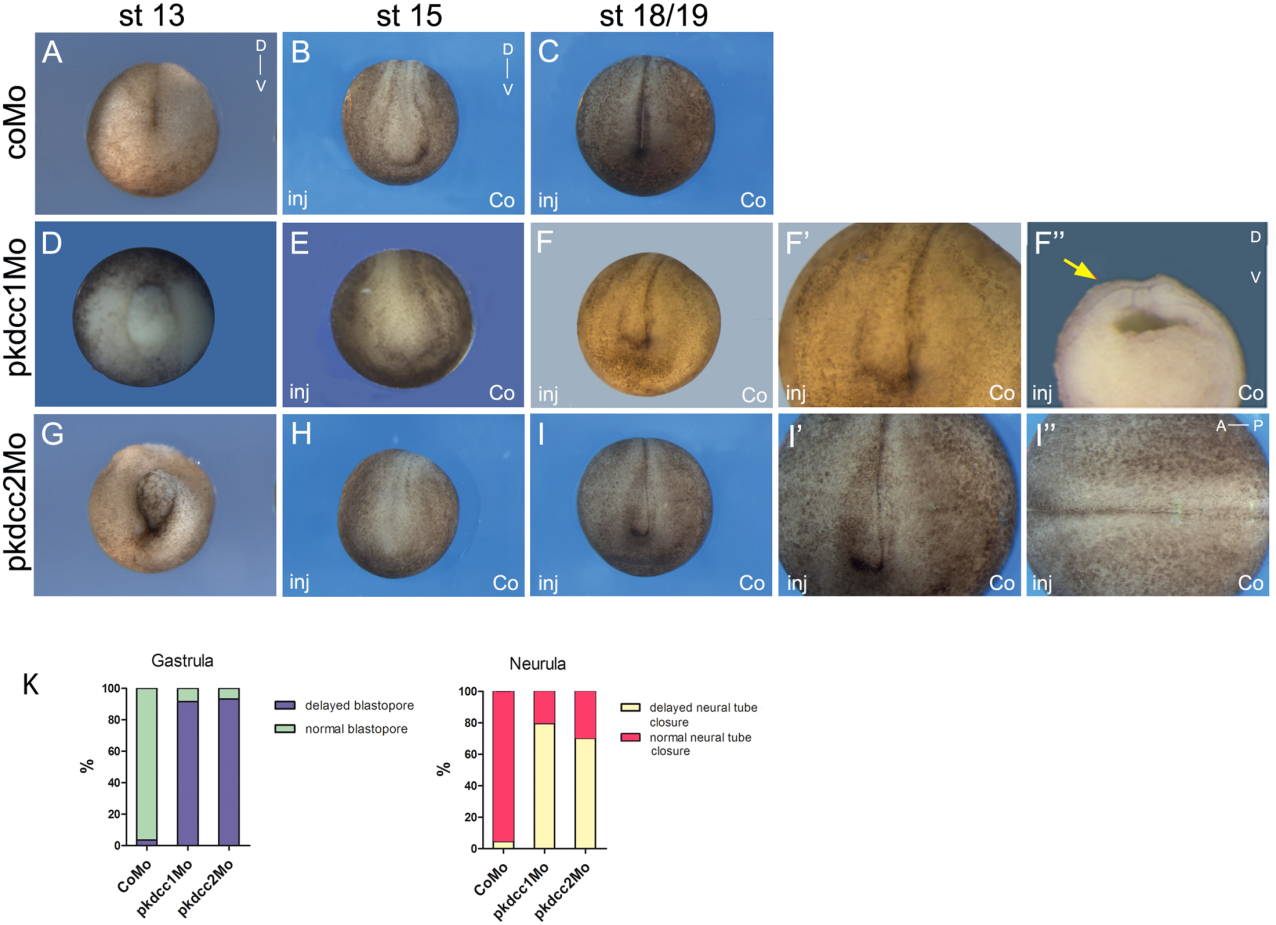
*In vivo* requirement of Pkdcc1 and Pkdcc2 during early development. (A-I”) Four cell stages were injected either (A, D, G) dorsally or (B-C, E-F”, H-I”) unilaterally with (D-F”) pkdcc1Mo, (G-I”) pkdcc2Mo or (A-C) coMo and analysed at (A, D, G) stage 13, (B, E, H) 15 or (C, F-F”, I-I”) 18/19. Injection of pkdcc1Mo and pkdcc2Mo caused (D, G) gastrulation and (E-F, H-I) neural tube closure defects that were not observed in (A-C) coMo injected embryos. (F’-F”, I’-I”) Magnification of the stage 18/19 pkdcc1Mo and pkdcc2Mo unilaterally injected embryos. Yellow and red arrows show the delay of neural fold formation. A, anterior; P, posterior; D, dorsal; V, ventral; inj, injected side; co, uninjected side.

**Fig 5:**
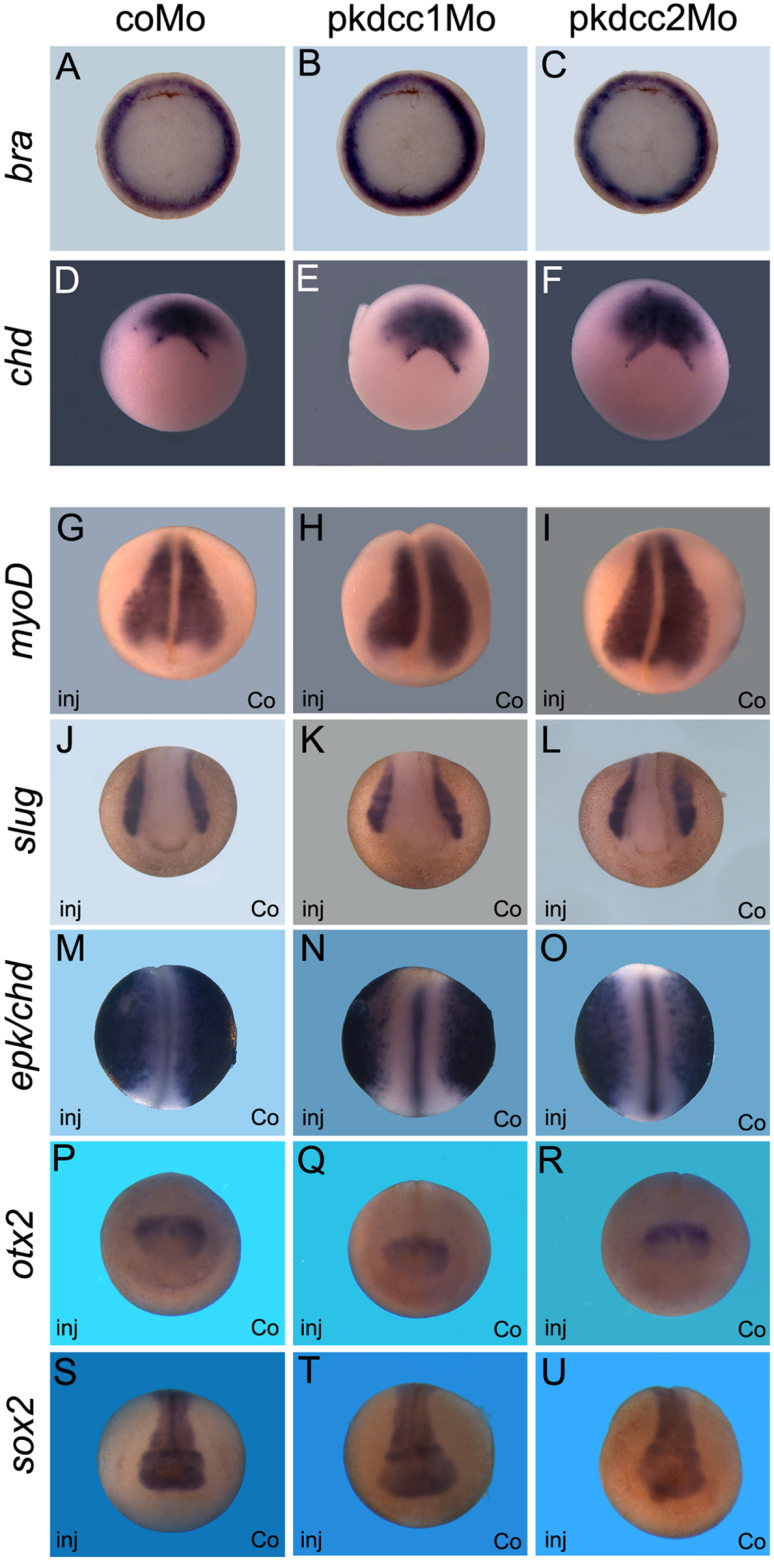
Absence of Pkdcc1 and Pkdcc2 does not change cell fate of mesoderm and neural tissues. Whole mount *in situ* hybridization for *Xbra* (A-C) and *chd* (D-F) of embryos injected with CoMo (A, D), pkdcc1Mo (B, E) or pkdcc2Mo (C, F) in the dorsal blastomeres. Whole mount *in situ* hybridization for *myoD* (G-I), *slug* (J-L), *epk/chd* (M-O), *otx2* (P-R) and *sox2* (S-U) of embryos injected with CoMo (G, J, M, P, S), pkdcc1Mo (H, K, N, Q, T) or pkdcc2Mo (I, L, O, R, U) in the right side. (A-F) Vegetal view of gastrula stage embryos with dorsal to the top. (G-U) Anterior view of embryos from late gastrula (G-I) to neurula stages (J-U) with dorsal to the top. inj, injected side; co, uninjected side.

The effect of the absence of Pkdcc1 and Pkdcc2 during embryonic development was assessed by microinjection of pkdcc1Mo, pkdcc2Mo or coMo (Control Morpholino) on both dorsal blastomeres of *X*. *laevis* embryos at 4-cell stage. At late gastrula stages, pkdcc1Mo (n = 118, 91.5%) or pkdcc2Mo (n = 120, 93.3%) injected embryos (Fig 4D and 4G) displayed an impaired closure of the blastopore that was not observed in the coMo injected embryos (n = 115, 3.5%; Fig 4A). Hemi-sectioned *pkdcc1* and *pkdcc2* morphant embryos with blastopore closure defects showed that, in the absence of Pkdcc1, the bottle cells, cells that undergo apical constriction and transform from cuboidal to flask-shaped inducing the formation of blastopore groove [35], are not well formed. (S1B and S1B’ Fig compared with S1A and S1A’ Fig). In contrast, in *pkdcc2* morphants, the bottle cells display typical flask-shape and are polarized (S1C and S1C’ Fig compared with S1A and S1A’ Fig). Moreover, we found that inactivation of *pkdccs* had no effect on mesoderm specification, since the absence of Pkdcc1 or Pkdcc2 has no effect on both *bra* and *chd* expression (Fig 5A–5F). The phenotype of pkdcc morphants overlapped the ones of PCP-deficient embryos, such as *syndecan 4* and *daam 1* morphants [36, 37]. This suggested that Pkdcc1 and Pkdcc2 could be involved in PCP signaling.

Both pkdcc1Mo and pkdcc2Mo phenotypes were rescued by the co-injection of the *pkdcc1* and *pkdcc2* mRNA mutated in the antisense morpholino oligonucleotide binding site *pkdcc1(mut)* (n = 85, 21.2%) and *pkdcc2(mut)* (n = 87, 22.9%), respectively, indicating that the obtained phenotype is specifically caused by the knockdown of Pkdcc1 or Pkdcc2 (S2 Fig).

In order to better evaluate the effects of Pkdcc1 and Pkdcc2 depletion, 4-cell stage embryos were unilaterally injected with pkdcc1Mo, pkdcc2Mo or coMo, in order to the uninjected side serve as internal control. At early neurula stages, while coMo injected embryos (n = 199, 4.5%) developed with no unusual phenotype (Fig 4B and 4K), pkdcc1Mo (n = 181, 79.6%) and pkdcc2Mo (n = 192, 70.3%) injected embryos displayed a delay in neural tube closure (Fig 4E, 4H and 4K). At this stage, on the pkdcc1Mo (or pkdcc2Mo) injected side, the embryo fails to form a well-defined neural fold. Interestingly, the neural folds seemed to be broader and more widely apart when compared with the non-injected side or with the coMo injected embryos. In addition, these phenotypes observed on the *pkdcc1* and *pkdcc2* morphants were more pronounced at stage 18/19 (Fig 4F and 4I compared with Fig 4C). The uninjected side developed normally being the neural folds placed already at, or very closely to, the dorsal midline (Fig 4C), while on the pkdcc1Mo (or pkdcc2Mo) injected side, the neural folds were still far from the midline (Fig 4F, 4F” and 4I, red and yellow arrows). These phenotypes were, once again, rescued by co-injection of *pkdcc1(mut)* (n = 89, 29.2%) and *pkdcc2(mut)* mRNA (n = 86, 25.6%), respectively (S2 Fig). In addition, co-downregulation of both Pkdcc1 and Pkdcc2 led to similar but more severe defects (data not shown). In hemi-sections of neurula staged embryos, in the absence of Pkdcc1, despite the enlarged neural crest and endoderm, it is possible to observe that the cells of presomitic mesoderm (PSM) are not well formed or organized. (S1D and S1D’ Fig). However, this is not observed in the absence of Pkdcc2. These morphants, despite the delay in the neural tube closure, only display an enlargement of the endodermal tissue.

Moreover, the observed phenotypes were not an indirect effect by loss of cell specification. By whole-mount *in situ* hybridization, we observed that cell fate markers of mesoderm (*bra*, *chd* and *myoD*), neural plate (*sox2*) and neuroectoderme (*sox2* and *otx*2), notochord (*chd*) and neural crest (*slug*) were not altered in Pkdcc1 and Pkdcc2 knocked-down embryos (Fig 5G–5U). Together, these results suggest that the absence of Pkdcc1 or Pkdcc2 cause defective cell movements.

**Fig 6:**
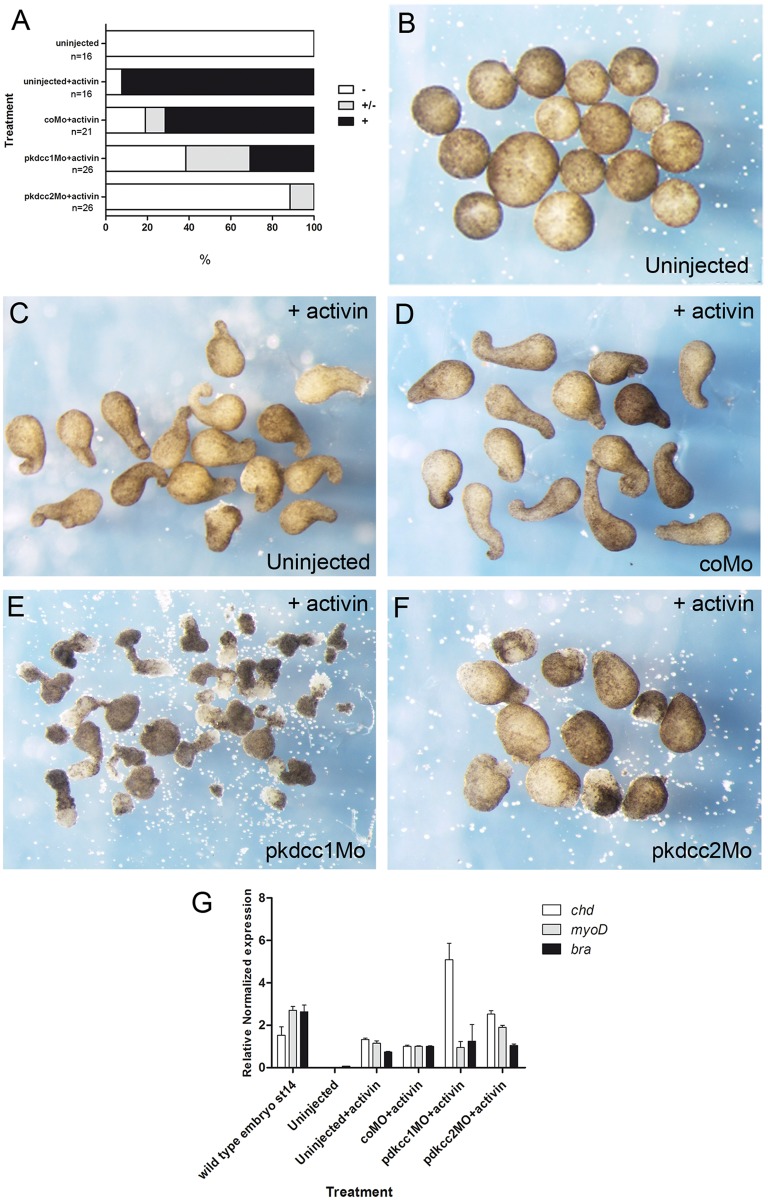
Effect of Pkdccs downregulation on convergent extension movements. (A) the percentage of elongated animal caps. -, no elongation; +/- partial elongation; +, strong elongation. (B) Uninjected animal caps without activin treatment. Uninjected (C) or injected animal caps with (D) coMo, (E) pkdcc1Mo or (F) pkdcc2Mo treated with activin. (G) qPCR analysis of mesodermal markers *chd*, *myoD* and *xbra* expression on wild type embryos at st 14 and uninjected or coMo, pkdcc1Mo or pkdcc2Mo injected animal caps in the presence of activin.

We assessed these defective cell movements by observing changes in the morphology of animal caps stimulated by activin. Uninjected animal caps, likewise coMo injected ones, elongated and underwent typical changes in morphology (Fig 6A–6D) [38]. The injection of pkdcc2Mo, inhibited the elongation of the animal caps (Fig 6A and 6F), confirming the defects observed previously in convergent extension during *pkdcc2* morphants development (Fig 4). Interestingly, the animal caps injected with pkdcc1Mo elongated, however an extensive cell spreading occurred in these explants and the length of elongation on half of the elongated animal caps was significantly reduced compared to uninjected or coMo injected animal caps (Fig 6F). In addition, in all animal caps subjected to activin treatment, the mesoderm was induced properly, indicating that the observed phenotypes are due to defective cell movement and not mesoderm specification (Fig 6G). Altogether, these results complement the *in vivo* results observed during *pkdcc* morphant development, where an inappropriate convergent extension was observed (Fig 4), suggesting a role for Pkdcc1 and Pkdcc2 in PCP pathway.

### Pkdcc1 and Pkdcc2 promote recruitment of Dishevelled to the plasma membrane through DEP domain

**Fig 7:**
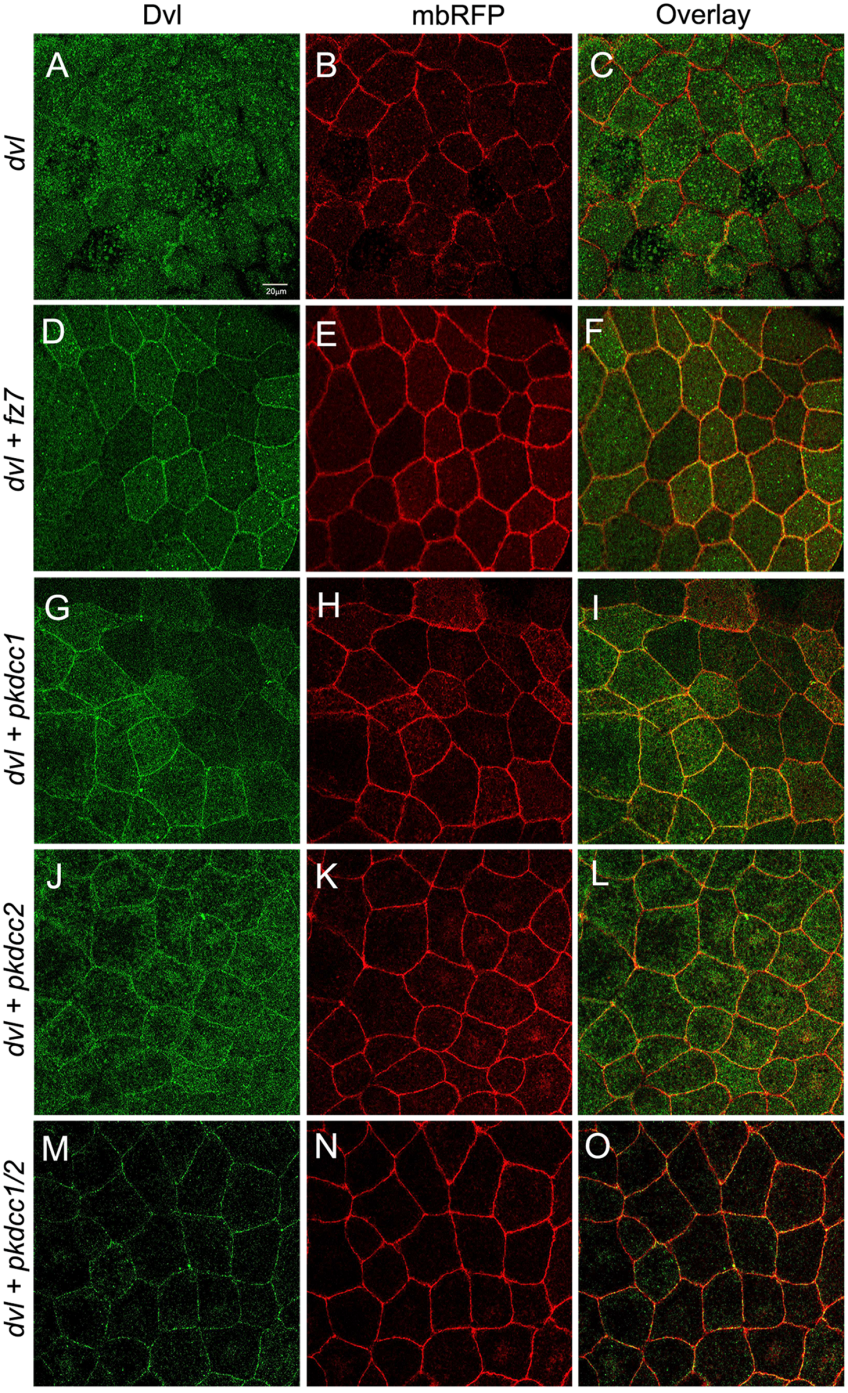
Both Pkdcc1 and Pkdcc2 promote the recruitment of Dvl to the plasma membrane. (A-O) Embryos were injected with the indicated mRNAs, the ectodermal explants were extracted and Dvl.GFP localization was observed by confocal microscopy. Dvl tagged with GFP (green) is shown on the left panel, the membrane bound RFP (mbRFP, red) is shown on the middle panel and the merge pictures are shown on the right panel. (A-C) Dvl.GFP is localized in the cytoplasm of animal cap cells injected with 300 pg of *dvl*.*GFP* mRNA. (D-F) When 300 pg of *dvl*.*GFP* mRNA are co-injected with 150 pg of *fz7* RNA, Dvl.GFP is recruited to the membrane. (G-I) Co-injection of 500 pg *pkdcc1* RNA and 300 pg of *dvl*.*GFP* mRNA leads to membrane recruitment of Dvl.GFP as well as (J-L) the co-injection of 500 pg of *pkdcc2* mRNA and 300 pg of *dvl*.*GFP* mRNA. (M-O) the same membrane localization of Dvl.GFP is observed when 300pg of *dvl*.*GFP* mRNA are co-injected with 500 pg of each *pkdcc1* and *pkdcc2* mRNAs.

Dishevelled (Dvl) is a protein involved in both canonical and non-canonical Wnt signaling, which regulates neural convergent extension [39, 40]. Moreover, the recruitment of Dvl into Frizzled receptor complexes at one cell edge is required for PCP signaling [41]. Since, the knockdown of both Pkdcc1 and Pkdcc2 disturbs the closure of the neural tube and likely the PCP signaling, we investigated if these proteins were involved in the recruitment of Dvl to plasma membrane. To test this, we used animal cap assays, like it was previously reported to study the recruitment of Dvl to the plasma membrane by Fz [42–47] Therefore, two-cell stage embryos were injected with different combination of mRNAs into the animal pole. At blastula stages, the animal caps were explanted and cultured until gastrula stages. At this stage, the protein localization was investigated by confocal microscopy. The results showed that Dvl.GFP is predominantly localized in the cytoplasm (Fig 7A–7C), but is recruited to the plasma membrane when co-expressed with Fz7 (Fig 7D–7F). Interestingly, the co-expression of Dvl.GFP with Pkdcc1, with Pkdcc2 or with both proteins also promoted the recruitment of Dvl to the plasma membrane (Fig 7G–7O).

**Fig 8:**
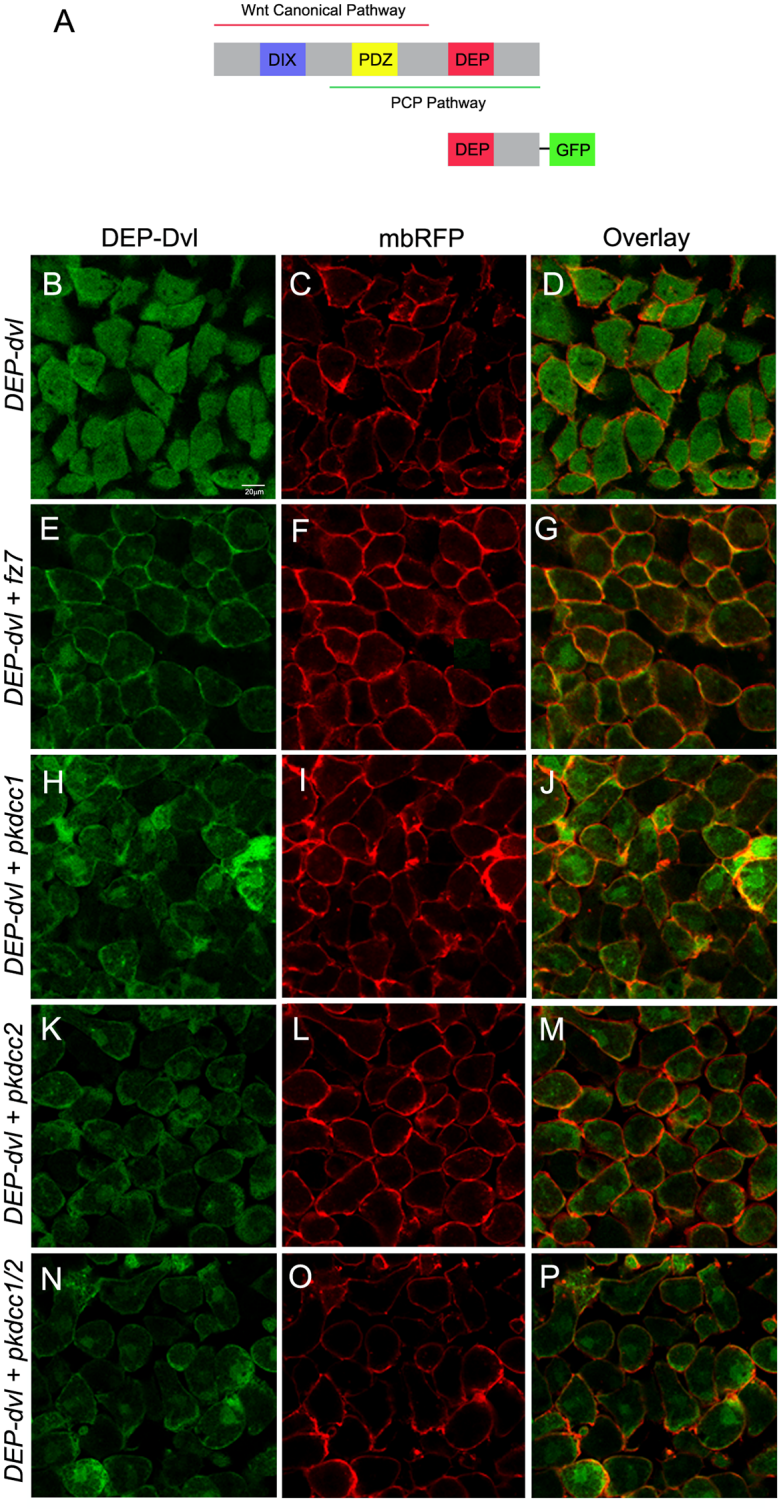
Pkdcc1 and Pkdcc2 promote the recruitment of Dvl to the plasma membrane through DEP domain. (A) Schematic representation of Dvl domains involved in both Wnt canonical and non-canonical signaling pathways, and DEP.Dvl construct containing the DEP domain of Dvl fused to GFP reporter. (B-P) Embryos were injected with the indicated RNAs, the ectodermal explants were extracted and DEP.Dvl localization was observed by confocal microscopy. DEP domain tagged with GFP (green) is shown on the left panel, the membrane bound RFP (mbRFP, red) is shown on the middle panel and the merged pictures are shown in the right panel. (B-D) DEP.Dvl is localized in the cytoplasm of cells when is overexpressed alone but is recruited to the plasma membrane when co-overexpressed with *fz7* (E-G). Co-injection of *DEP*.*dvl* and *pkdcc1* (H-J), *pkdcc2* (K-M) or both simultaneously (N-P) leads to the membrane recruitment of DEP.Dvl.

Dvl is a protein that consists essentially in three conserved domains, DIX, PDZ and DEP (Fig 8A) [48, 49]. The DIX domain was previously shown to be involved in Wnt canonical signaling, the DEP domain in the PCP pathway and the PDZ domain in both Wnt canonical and non-canonical signaling pathways [26, 50–52]. Because our results suggest that both Pkdcc1 and Pkdcc2 could be involved in PCP pathway, we tested if the DEP domain of Dvl is required for the membrane-recruitment promoted by Pkdcc proteins. To evaluate this hypothesis, we used a construct in which only the DEP domain of Dvl was fused to the GFP reporter (DEP.Dvl, Fig 6A) to redo the previous experiment. Consistently, the DEP.Dvl was predominantly detected in the cytoplasm of the ectodermal cells, when expressed alone (Fig 8B–8D). However, this recombinant protein localizes in the plasma membrane when co-expressed with Fz7 (Fig 8E–8G), like it was previously described [26], with Pkdcc1 (Fig 7H–7J), with Pkdcc2 (Fig 7K–7M) or when both Pkdcc1 and Pkdcc2 were co-expressed with DEP.Dvl (Fig 7N–7P). These results indicate that both Pkdcc1 and Pkdcc2 promote the recruitment of Dvl to the plasma membrane throughout DEP domain.

### Pkdcc1 and Pkdcc2 regulate ATF2 expression

**Fig 9:**
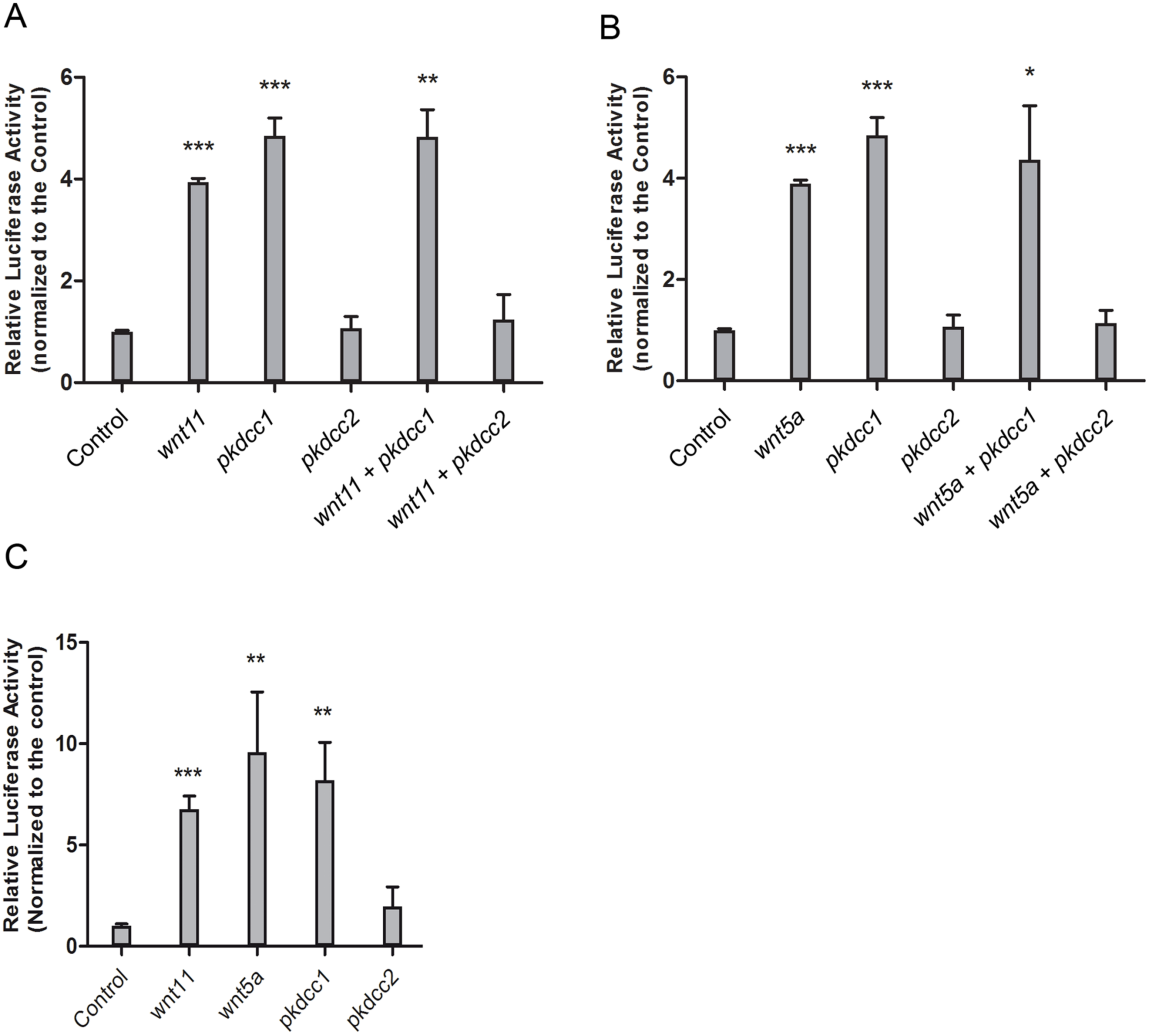
Pkdcc1 is an inducer of PCP signaling while Pkdcc2 is a repressor. (A, B) HEK293T cells were transfected with the indicated constructs in addition to an ATF2 luciferase reporter construct and a β-galactosidase expression vector. Luciferase activity was measured 48h after transfection and normalized with β-galactosidase activity. Each experiment was carried out in triplicates and error bars represent the standard deviation. (C) *X*. *laevis* embryos were injected radially at two cell stage with the indicated constructs in addition to an ATF2 luciferase reporter construct and a β-galactosidase expression vector. Luciferase activity was measured at gastrula stages (st11) and normalized with β-galactosidase activity. Error bars represent the standard deviation of the mean.

Since both Pkdccs were able to promote the recruitment of the Dvl to the cellular membrane, we wanted to better understand the involvement of these molecules in PCP signaling. To test this, we performed luciferase assays using ATF2 reporter that monitors JNK dependent PCP signaling [53]. *pkdcc1* and *pkdcc2* coding sequence containing plasmids were transfected in HEK293T cells alone or together with *wnt5a* or *wnt11* and the cells were allowed to grow for 48h. *β-galactosidase* plasmid was also transfected and used for standardization. This assay showed that Pkdcc1 alone is able to induce the expression of *Atf2-luc*, and the activation of non-canonical Wnt signaling (Fig 9A and 9B). Curiously and contrary, Pkdcc2 is not able to activate *Atf2* expression, inhibiting the normal activation of JNK dependent non-canonical Wnt downstream of Wnt11 or Wnt5a (Fig 9A and 9B). The same results were obtained by *in vivo* experiments, the overexpression of *pkdcc1* mRNA in *X*. *laevis* embryos was able to induce the expression of *Atf2-luc* but, in contrast, the overexpression of *pkdcc2* that was not able to induce *Atf2-luc* expression (Fig 9C).

**Fig 10:**
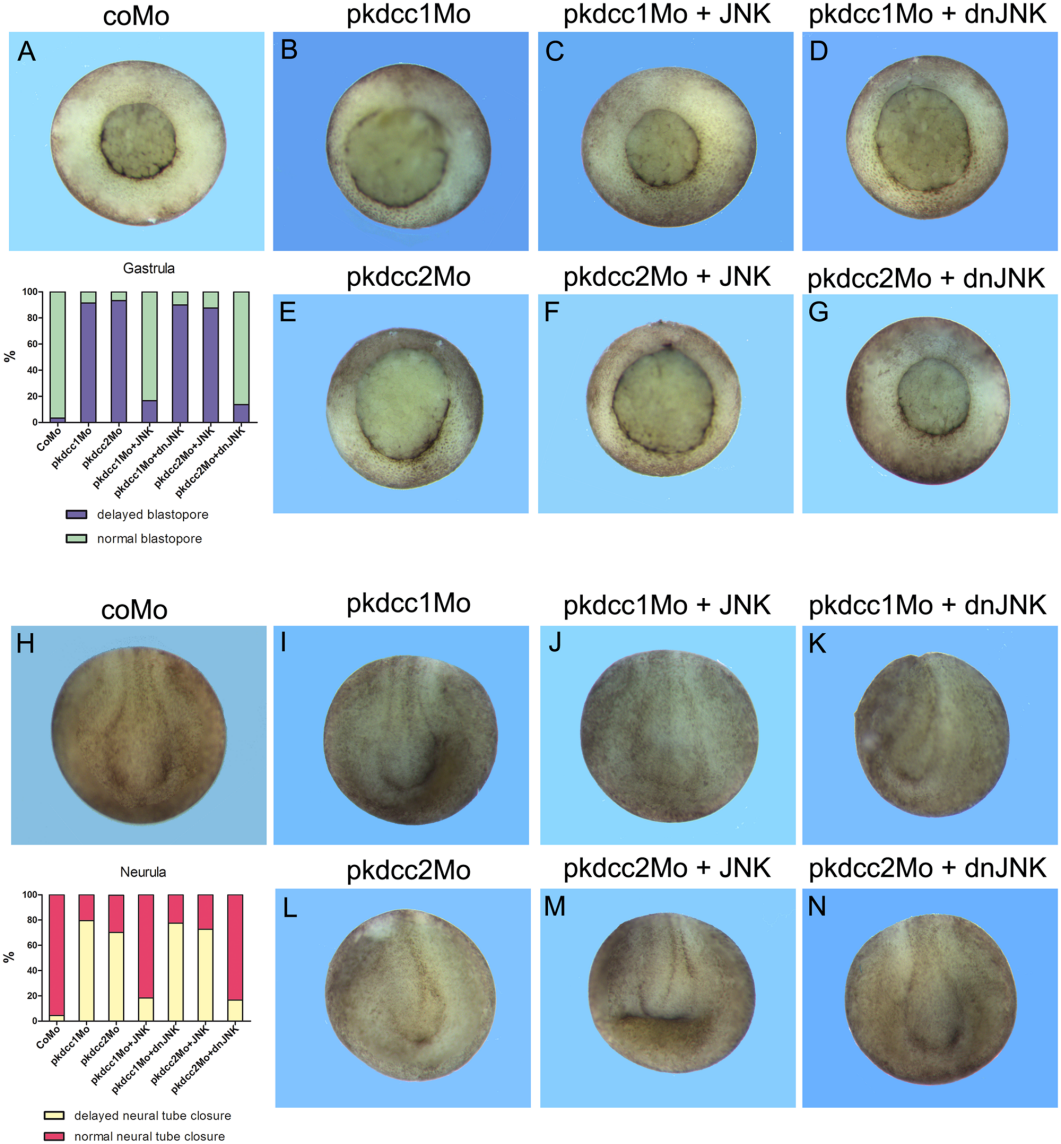
Rescue of pkdcc1Mo and pkdcc2Mo phenotype by JNK and dnJNK, respectively. (A- G) Four cell stage embryos were injected dorsally with coMo (A), pkdcc1Mo (B-D) or pkdcc2Mo (E-G) and incubated until blastopore closure. The pkdcc1Mo phenotype (delay in blastopore closure) was rescued by injection with *JNK* mRNA (C) but not with *dominant negative JNK* (*dnJNK*) mRNA(D). Contrary, pkdcc2Mo phenotype was rescued by the injection with the *dnJNK* mRNAbut not with *JNK* mRNA. (H-N) Four cell stage embryos were unilaterally injected with coMo (H), pkdcc1Mo (I-K) or pkdcc2Mo (L-N) and incubated until neural tube closure. Once again, the phenotype observed in the absence of Pkdcc1 (delay in neural tube closure) was rescued by the overexpression of *JNK* mRNA (J) but not of *dnJNK* mRNA (K). Contrary, pkdcc2Mo phenotype was rescued by co-injection of *dnJNK* mRNA (M) but not of *JNK* mRNA (N).

To confirm these results we performed rescue experiments in which Pkdcc1 and Pkdcc2 knockdown phenotypes were retrieved by JNK and dominant-negative JNK (dnJNK), respectively. With this purpose, to rescue the phenotype of Pkdcc1 absence, 4-cell stage embryos were injected in the two dorsal blastomeres or unilaterally, at the right side with coMo, pkdcc1Mo alone, or co-injected with *jnk* or *dnjnk* mRNA, and allowed to grow until gastrula and neurula stages. The results showed that pkdcc1Mo phenotype was rescued both in gastrula and neurula stages by the overexpression of *jnk* (Fig 10A–10C and 10H–10J). As expected, the overexpression of *dnjnk* could not rescue the pkdcc1Mo phenotype (Fig 10D and 10K).

To rescue the phenotype of Pkdcc2 absence, once again 4-cell stage embryos were injected in the two dorsal blastomeres or unilaterally, at the right side with coMo, pkdcc2Mo alone, or co-injected with *jnk* or *dnjnk* mRNA, and allowed to grow until gastrula and neurula stages. The results showed that, contrary to pkdcc1Mo, pkdcc2Mo phenotype was rescued by the co-injection of *dnjnk*, but not *jnk* mRNA, in both gastrula and neurula stages (Fig 10E–10H and 10L–10N). These results support the idea that both Pkdcc1 and Pkdcc2 are involved in the regulation of JNK dependent non-canonical Wnt/PCP signaling, leading to the activation or repression of this pathway, respectively.

## Discussion

*pkdcc1* and *pkdcc2* are two genes that display a very dynamic expression, detectable from gastrula stages onwards. During gastrula stages, *X*. *laevis pkdcc* genes are expressed in the anterior dorsal endoderm (ADE) and, later on, in the involuting mesendoderm. Curiously, the mouse orthologs of these *Xenopus* genes is expressed in the topological equivalent mouse AVE region [23, 30, 31], suggesting that the expression of these genes family is evolutionary conserved [1–3]. In addition, *pkdcc1* is also expressed in the dorsal blastopore lip and in the dorsal neural ectoderm. At later stages, *pkdcc1* is expressed in tissues including eyes, isthmus, foregut and notochord. On the other hand, tissues expressing *pkdcc2* include the neural folds, eyes, otic vesicle and notochord. These very dynamic expression patterns observed in both *X*. *laevis pkdcc* genes suggested that they participate in multiple roles during embryonic development.

These roles were investigated by injection of Pkdcc1 and Pkdcc2 antisense morpholino oligonucleotides [33]. The reduction of each Pkdcc protein interferes with blastopore and neural tube closure during early *X*. *laevis* development. These phenotypes are usually related with defective cell migration, namely convergent extension (CE). For example, both overexpression and knockdown of *syndecan-4*, knockdown of Wnt11 or Wnt5, or and overexpression of *Xdd1* cause defects in CE [36, 54].

Our animal cap assays showed that Pkdcc2Mo inhibits CE movements induced by activin. This results is in agreement with overexpression of Wnt5a (that, like the absence of Pkdcc2, induces JNK activation) in animal caps treated with activin, where CE movements were also inhibited [55]. On the other hand, Pkdcc1Mo was not able to fully inhibit these movements, although, the animal caps presented an extensive cell spreading, suggesting a decrease in cell-cell adhesion. This is in agreement with previous reports indicating that the appropriate activation of JNK is necessary to cell-cell adhesion and concomitantly for correct convergent extension [38, 55].

In the vertebrate embryo, CE movements are those movements responsible for the elongation of the anterior-posterior axis while the mediolateral axis narrows [56]. Hemisections of the knockdown embryos during gastrula stages showed that, in the absence of Pkdcc1, the bottle cells are not properly formed. The blastopore groove is formed when bottle cells undergo apical constriction and transform from cuboidal to flask-shaped [35]. In the absence of Pkdcc2, the bottle cells are well formed. This is in accordance with our data, since only Pkdcc1 and not Pkdcc2 is expressed in the dorsal blastopore lip, the region where bottle cells start to be formed. *Wnt5a*, a gene of non-canonical Wnt signaling was also implicated in the formation of bottle cells, since its overexpression induced ectopic bottle cells formation, whereas its down regulation supresses bottle cells formation in *X*. *laevis* [57]. In other species, some members of PCP pathway were also related with bottle cell formation suggesting a role of PCP signaling in the process of bottle cells formation [35, 58–62]. This process is completely independent of Wnt canonical signaling, since the interruption of this pathway inhibits endoderm cell fate specification but not bottle cells formation [63].

During neurulation, the neural tube closure requires neural fold elevation, bending and conversion. Our loss-of-function experiments showed that the downregulation of each Pkdcc depletion cause neural tube closure defects, most probably due to defective CE movements since during neurulation the midline CE is necessary to reduce the distance between the two forming neural folds allowing them to meet and fuse the forming the neural tube [64, 65].

Hemisection of knockdown embryos showed that in the absence of Pkdcc1, besides the delay in neural tube closure, both neural crest and endoderm were enlarged and the presomitic mesoderm (PSM) was not well polarized. The cells of PSM were not elongated as usual, but they presented a round shape. These defects were not observed in the absence of Pkdcc2, since in these embryos besides neural tube closure defects, only the endoderm was enlarged.

The type of defects observed here, in the absence of both Pkdcc1 or Pkdcc2 during gastrulation and neurulation, i.e., impaired CE and defects in neural tube closure, are largely associated with disruption of Wnt/PCP signaling caused by mutations in core PCP pathway proteins and other PCP regulatory proteins [36, 64, 66–69]. This suggests that Pkdcc1 and Pkdcc2 are involved in the regulation of the proper levels of PCP signal during, at least, these two morphogenetic processes.

Nevertheless, the defects observed in neural tube closure can also be associated to the disruption of other signaling pathways, such as Hedgehog pathway or defects in actin cytoskeleton [49]. Disruption of core components of the PCP pathway, like Dvl, results in posterior neural tube closure defects, while disruption of the Hedgehog signaling or the actin cytoskeleton results in anterior neural tube defects [49, 64]. Recently, Probst *et al*, showed that *Pkdcc* genetically interacts with *Gli3*, a member of Hedgehog signaling during the formation of long bones in mouse embryos. They show that the knockout of *Pkdcc* leads to the formation of shorter long bones that is aggravated by the double knockout of *Gli3* and *Pkdcc* [4]. Although not shown here, a relationship between both Pkdcc1 and Pkdcc2 and Hedgehog signaling cannot be excluded. Nevertheless, the authors also suggest an alternative model where PKDCC could also modulate Wnt signaling, since Wnt5a inactivation also affects the formation of hypertrophic chondrocytes [4]

Here, we show that bothPkdcc1 and Pkdcc2 are able to promote the recruitment of Dvl to the plasma membrane through DEP domain. There is a wealth of evidences that recruitment of Dvl into Frizzled receptor complexes at one cell edge is required for PCP signaling [41, 43, 70, 71]. This suggests us that both Pkdcc proteins are involved somehow in the recruitment of Dvl to the plasma membrane and therefore in PCP signaling. Nevertheless, we think that the recruitment of Dvl to the plasma membrane by both Pkdcc proteins is not directly since we could never observed their presence in the plasma membrane (data not shown). Instead, like their mouse ortholog, both Pkdcc1 and Pkdcc2 proteins are localized in the Golgi apparatus (S3 Fig). Kinoshita *et al* suggested that the Golgi localization of mouse PKDCC is important for its role in protein secretion. They showed that the protein transport to the plasma membrane is PKDCC-level dependent in NIH3T3 cells [3]. Since both *X*. *laevis* Pkdccs are also localized in the Golgi apparatus, we hypothesized that these Pkdccs could have a similar function in PCP signaling regulating somehow the transport of PCP core proteins for its final destination. Further experiments are required to address this in depth.

Indeed, our results showed that Pkdcc1 is able to induce a luciferase reporter under the control of *Atf2* promoter. This reporter was shown to respond to PCP components such as Wnt, Fz, Dvl [53], and was used has a readout of JNK dependent PCP pathway. Surprisingly, Pkdcc2 is not able to induce the activation of this reporter but, contrary, it is able to inhibit its normal activation by Wnt11 or Wnt5a. These results suggest that, despite the similarities observed between their phenotypes, Pkdcc1 and Pkdcc2 have different roles in JNK dependent PCP signaling pathway. These experiments were supported by the fact that we are able to rescue the phenotypes obtained in the absence of Pkdcc1 or Pkdcc2, during both gastrulation and neurulation, by overexpressing JNK or a dominant negative form of JNK, respectively.

In summary, our results show for the first time that two members of PKDCC family, *X*. *laevis* Pkdcc1 and Pkdcc2 proteins are involved in the regulation of JNK dependent PCP signaling.

## Supporting Information

S1 Fig Hemi-sections of Pkdcc1 and Pkdcc2 knockdown embryos during gastrula and neurula stages. (A-C) Hemi-section of *X*.*laevis* embryos injected dorsally with CoMo (A), pkdcc1Mo (B) or pkdcc2Mo (C) at gastrula stage. Auto-fluorescence of the embryo was observed by confocal microscopy. Dorsal to the left and animal to the top (A’-C’). Schematic representation of embryos A-C, respectively. (D, E) Hemisection of *X*.*laevis* embryos injected unilaterally with pkdcc1Mo (D) or pkdcc2Mo (E). Auto-fluorescence of the embryo was observed by confocal microscopy. Dorsal to the top. (D’, E’) Schematic representation of embryos D, E, respectively. bc, bottle cells; idm, involuting dorsal mesoderm; dbl, dorsal blastopore lip; sm, presomitic mesoderm; nc, neural crest; e, endoderm.
(TIF)

S2 Fig Rescue of pkdcc1Mo and pkdcc2Mo phenotype by *pkdcc1(mut)* and *pkdcc2(mut)* mRNAs, respectively. (A-E) Four cell stage embryos were injected dorsally with coMo (A), pkdcc1Mo (B) or pkdcc2Mo (D) and incubated until blastopore closure. The pkdcc1Mo phenotype was rescued by co-injection with 1ng of *pkdcc1(mut)* mRNA (C) and pkdcc2Mo phenotype was rescued by the co-injection with the 1ng of *pkdcc2(mut)* mRNA. (F-J) Four cell stage embryos were unilaterally injected with pkdcc1Mo (G), pkdcc2Mo (I) or coMo (F) and incubated until neural tube closure. Once again, the phenotype obtained by the absence of Pkdcc1 was rescued by the overexpression of *pkdcc1(mut)* mRNA (H) and pkdcc2Mo phenotype was rescued by co-injection of *pkdcc2(mut)* mRNA (J). n is the number of injected embryos and the percentage stands for the embryos with the observed defect.
(TIF)

S3 Fig Co-localization of Rab8, Pkdcc1 and Pkdcc2 in the Golgi apparatus. Transfection of HEK293T cells with (A-C) *Rab8*.*GFP* (50 ng) and *Pkdcc1*.*HA* (1 μg) or (D-F) with *Rab8*.*GFP* (50 ng) and *Pkdcc2*.*myc* (1 μg). Immunofluorescence against HA (B) and myc (E) was performed. Overlay of Rab8 and Pkdcc1 (C) or Pkdcc2 (F) are represented in the right side of the panel.
(TIF)

S1 Table Primers used to cloning. (DOCX)

S2 Table Primers used for quantitative RT-PCR. (DOCX)
