# Supplementary figures and images for: Xenopus Pkdcc1 and Pkdcc2 Are Two New Tyrosine Kinases Involved in the Regulation of JNK Dependent Wnt/PCP Signaling Pathway
Source: PLoS One. 2015 Aug 13;10(8):e0135504. doi: 10.1371/journal.pone.0135504 (PMC4536202; doi:10.1371/journal.pone.0135504)

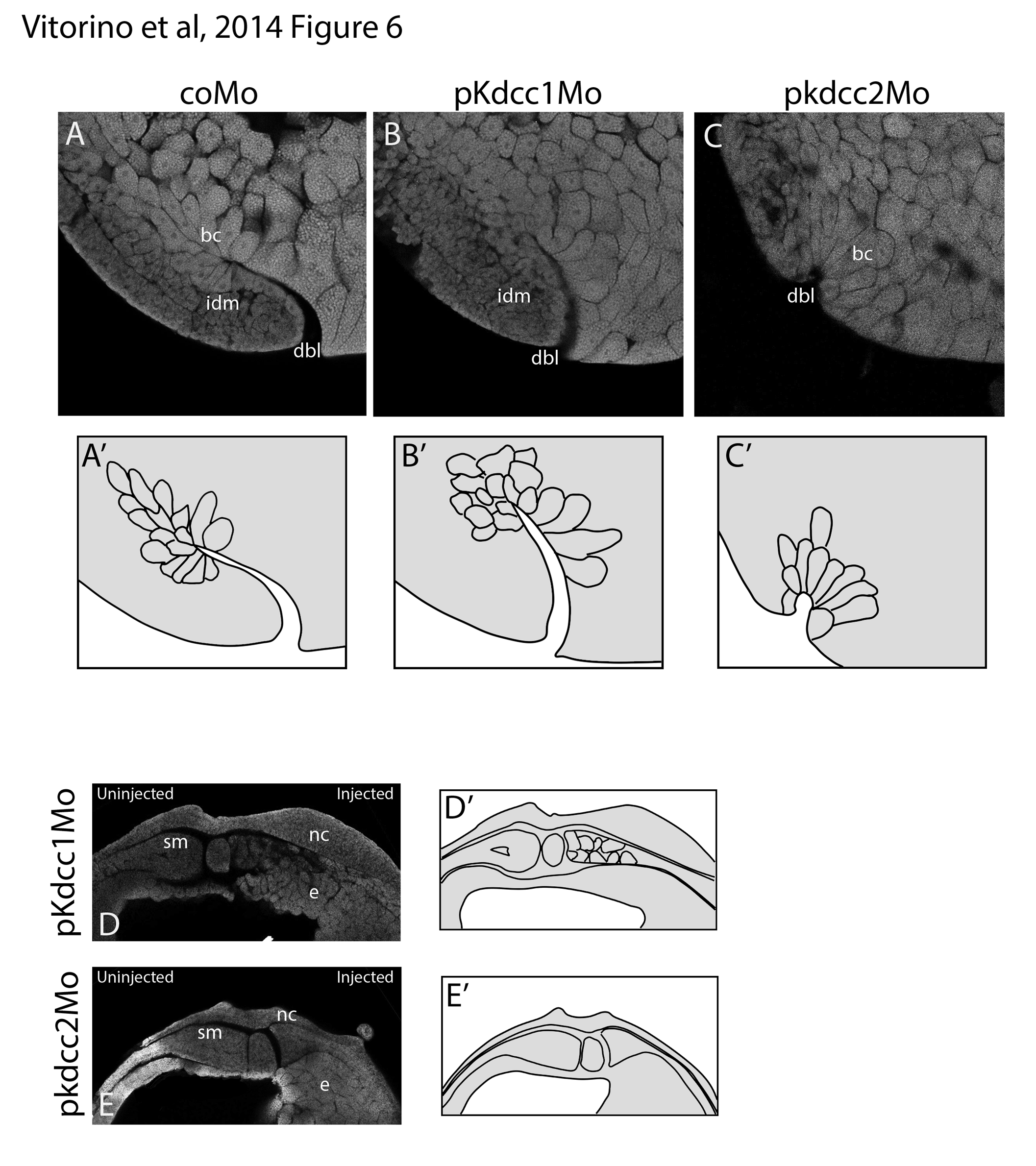

Supplement: S1 Fig — (A-C) Hemi-section of X.laevis embryos injected dorsally with CoMo (A), pkdcc1Mo (B) or pkdcc2Mo (C) at gastrula stage. Auto-fluorescence of the embryo was observed by confocal microscopy. Dorsal to the left and animal to the top (A’-C’). Schematic representation of embryos A-C, respectively. (D, E) Hemisection of X.laevis embryos injected unilaterally with pkdcc1Mo (D) or pkdcc2Mo (E). Auto-fluorescence of the embryo was observed by confocal microscopy. Dorsal to the top. (D’, E’) Schematic representation of embryos D, E, respectively. bc, bottle cells; idm, involuting dorsal mesoderm; dbl, dorsal blastopore lip; sm, presomitic mesoderm; nc, neural crest; e, endoderm. (TIF) [file pone.0135504.s001.tif]

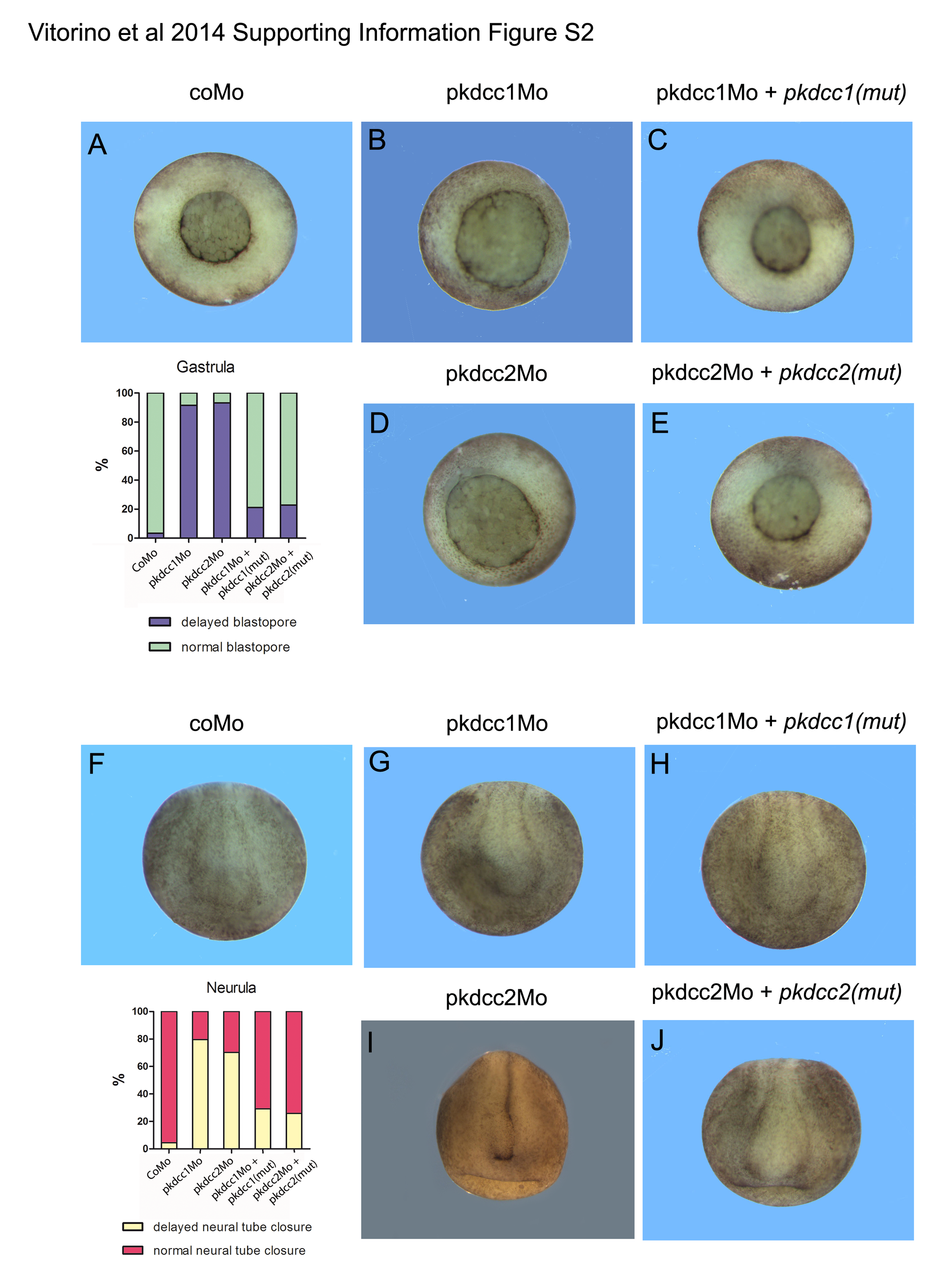

Supplement: S2 Fig — (A-E) Four cell stage embryos were injected dorsally with coMo (A), pkdcc1Mo (B) or pkdcc2Mo (D) and incubated until blastopore closure. The pkdcc1Mo phenotype was rescued by co-injection with 1ng of pkdcc1(mut) mRNA (C) and pkdcc2Mo phenotype was rescued by the co-injection with the 1ng of pkdcc2(mut) mRNA. (F-J) Four cell stage embryos were unilaterally injected with pkdcc1Mo (G), pkdcc2Mo (I) or coMo (F) and incubated until neural tube closure. Once again, the phenotype obtained by the absence of Pkdcc1 was rescued by the overexpression of pkdcc1(mut) mRNA (H) and pkdcc2Mo phenotype was rescued by co-injection of pkdcc2(mut) mRNA (J). n is the number of injected embryos and the percentage stands for the embryos with the observed defect. (TIF) [file pone.0135504.s002.tif]

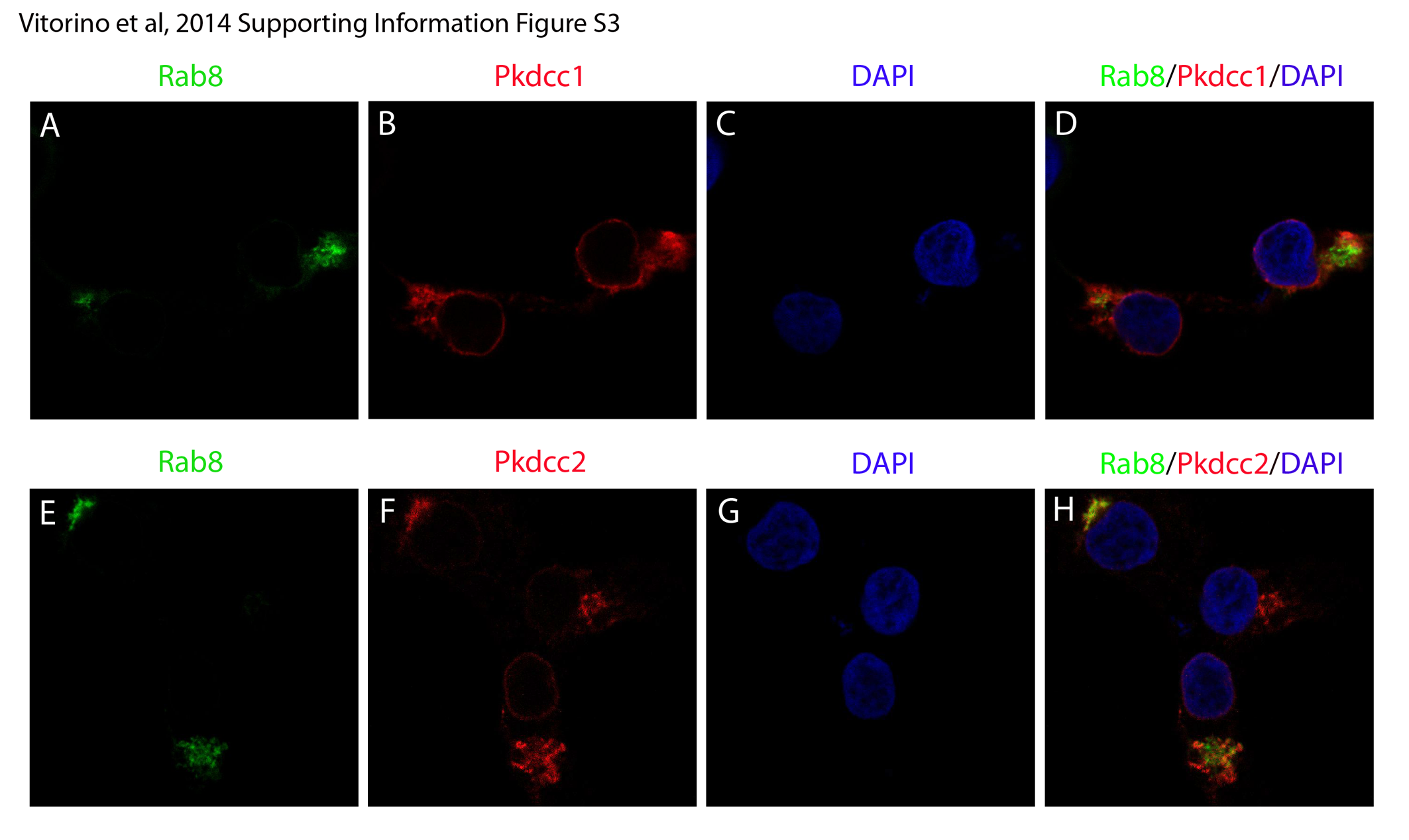

Supplement: S3 Fig — Transfection of HEK293T cells with (A-C) Rab8.GFP (50 ng) and Pkdcc1.HA (1 μg) or (D-F) with Rab8.GFP (50 ng) and Pkdcc2.myc (1 μg). Immunofluorescence against HA (B) and myc (E) was performed. Overlay of Rab8 and Pkdcc1 (C) or Pkdcc2 (F) are represented in the right side of the panel. (TIF) [file pone.0135504.s003.tif]
