# Supplementary material for: Xenopus Pkdcc1 and Pkdcc2 Are Two New Tyrosine Kinases Involved in the Regulation of JNK Dependent Wnt/PCP Signaling Pathway
Source: PLoS One. 2015 Aug 13;10(8):e0135504. doi: 10.1371/journal.pone.0135504 (PMC4536202; doi:10.1371/journal.pone.0135504)
Supplement: S1 Table — (DOCX) [file pone.0135504.s004.docx]

**Table S1** – Primers used to cloning.

| Primers for cloning (5’-3’) | | |
| --- | --- | --- |
| Name of primer | Forward | Reverse |
| pkdcc1 | TTTGGATCCAGTGATGAAGAACACC | GCAGATGGGAAGATGATCGATTTT |
| Pkdcc1HA | AATTCCCGTGTTTACATGAAAAGACCAAGTGACCTCAAAAAGTAT | CGATACTTTTTGAGGTCACTTGGTCTTTTCATGTAAACACGGG |
| Pkdcc2myc | TTTGGATCCCATCATGAGACGCAGG | aaaatcgatagcctgtcagtttcaggtacg-3’ |
