# Supplementary material for: Xenopus Pkdcc1 and Pkdcc2 Are Two New Tyrosine Kinases Involved in the Regulation of JNK Dependent Wnt/PCP Signaling Pathway
Source: PLoS One. 2015 Aug 13;10(8):e0135504. doi: 10.1371/journal.pone.0135504 (PMC4536202; doi:10.1371/journal.pone.0135504)
Supplement: S2 Table — (DOCX) [file pone.0135504.s005.docx]

| Primers for RT-PCR and qPCR (5’-3’) | | |
| --- | --- | --- |
| Name of primer | Forward | Reverse |
| pkdcc1 | GGGAGATGCACTGGCGTCAATG | TGCAGAACTCGGCAGCCTCTTC |
| pkdcc2 | ACTGCAAAGGCTTCAGCACC | TCGGTGTGGCTGATACAAGG |
| gapdh | GACCTGCCGCCTGCAGAA | GACTAGCAGGATGGGCGAC |
| odc | CAGCTAGCTGTGGTGTGG | CAACATGGAAACTCACACC |
| xnr3 | CGAGTGCAAGAAGGTGGACA | ATCTTCATGGGGACACAGGA |
| sia | AAGATAACTGGCATTCCTGAGC | GGTAGGGCTGTGTATTTGAAGG |
| β-catenin | AGATGCAGCAACTAAACAGGA | GTACTGCATTTTGAGCCATCT |
| chd | ACTGCCAGGACTGGATGGT | GGCAGGATTTAGAGTTGCTTC |
| bra | ttggagcccactggatgaaa | tgcggtcactgctatgaactgt |
| myoD | ccctgtttcaatacctcagacat | cgtgctcatcctcgttatgg |

**Table S2** – Primers used for quantitative RT-PCR.
